# Current Screens Based on the AlphaScreen™ Technology for Deciphering Cell Signalling Pathways

**DOI:** 10.2174/138920209787847041

**Published:** 2009-04

**Authors:** Saïd Taouji, Sophie Dahan, Roger Bossé, Eric Chevet

**Affiliations:** 1 Avenir, INSERM U889, Montreal, Qc, Canada; 2 Université Bordeaux 2, Bordeaux, France, Montreal, Qc, Canada; 3 PerkinElmer Biosignal, Montreal, Qc, Canada

**Keywords:** Signal transduction, signaling, Alphascreen™.

## Abstract

Global deciphering of signal transduction pathways represents a new challenge of the post-genomic era. However, for the majority of these signaling pathways the role(s), the function(s) and the interaction(s) of the signaling intermediates remain to be characterized in an integrated fashion. The global molecular study of cell signaling pathways and networks consequently requires sensitive, robust technologies which may allow in addition multi-parallel and highthroughput applications. The Alphascreen™ technology, relying on a bead-based homogenous approach, constitutes a valuable tool to detect and quantify a wide range of signaling events such as enzymatic activities or biomolecular interactions. In this article, we exhaustively review the literature and report the broad spectrum of Alphascreen™-based applications in the study of signal transduction pathways.

## 1). INTRODUCTION

Intracellular signaling involves a complex network of pathways that activate, link and coordinate various functions of the cell. The ability of intracellular signaling networks to integrate, process and distribute the information through the cell requires that individual signaling partners must interact with multiple effectors identified as inputs or outputs. In addition a significant number of non-proteinaceous entities (e.g. lipids, ions, metabolites) also participate in increasing the complexity of the above mentioned molecular signaling networks. Consequently, it becomes of interest to develop specific approaches to monitor, in parallel, a number, as large as possible, of events occurring in a homogenous cell population and which may reflect the activation of specific signaling cascades in precise cellular contexts.

The observation that, besides intrinsic enzymatic activities, proteins exert virtually all of their functions *via* interactions with other molecules – be they other proteins, nucleic acids, lipids, carbohydrates or small molecules – has driven the development of technologies to examine these macromolecular associations. The development of large-scale and versatile assay formats is therefore needed for monitoring enzymatic activities, modifications, interactions or the combination of those parameters for which there are very few direct methods available at present. The amplified luminescence proximity homogenous assay is an example of a technology developed for the biological sciences in the past few years that has followed a progression leading to a genomic scale use. The Alphascreen™ (AS) technology concept also proved to be remarkably malleable, with novel adaptations allowing for the detection of protein–DNA, protein–RNA, or protein–small molecule interactions, as well as protein–protein interactions that are dependent or not on post-translational modifications or which occur in different compartments of the cell. Indeed, in the past few years, a large number of high-throughput assays were developed to decipher cellular signaling pathways or to identify compounds that may modulate protein-protein interactions or enzymatic activities, respectively. Here we review the development of functional AS assays which will be providing an integrated understanding of cell signaling pathways.

## 2). THE ALPHASCREEN™ TECHNOLOGY – HISTORY, PRINCIPLES AND OVERALL DESCRIPTION

**Fig. (1):**
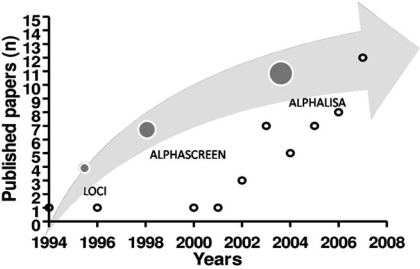
Schematic representation of the Alphascreen™ technology development time-line (gray arrow) placed in parallel to the related published papers (n=46).

Originally the technology that led to the development of AS, is a luminescent oxygen channeling immunoassay (LOCI). LOCI is a homogeneous bead-based immunoassay method capable of rapid, quantitative determination of a wide range of analytes including high and very low concentrations of large and small molecules, free (unbound) drugs, DNA, and specific IgM. In the mid 1990’s, a group at Syva/Dade Behring developed numerous assays for laboratory diagnostics purposes [1, 2]. In 1999 and since then, Perkin-Elmer has acquired the exclusive rights to develop the LOCI technology for research and drug screening applications. The novel trademarked technology named AlphaScreen™ was born. Since 1999, new homogenous assays appeared to measure several aspects of the expression/activation of signal transduction molecules, enzymatic activities, to screen for compounds or to quantify specific biomarkers (Fig. **1**).

These assays derived from LOCI technology, use latex particle pairs which are formed in the assays through specific binding interactions by sequentially combining the sample and two reagents. One particle contains a photosensitizer whereas the other contains a chemiluminescer. Irradiation causes the photosensitized formation of singlet oxygen species in the photosensitizer-containing bead. The singlet oxygen species transfer to a bound particle and activates the chemiluminescer, thereby initiating a delayed luminescence emission. The singlet oxygen species display a lifetime of approximately 4 µs which allows them to travel 200 nm in aqueous solutions.

**Fig. (2):**
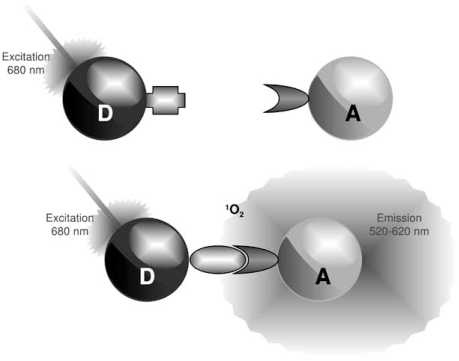
Principles of Alphascreen™.

Based on these principles, the AS technology has been developed in which Donor (photosensitizer) and Acceptor (chemiluminescer) microbeads can be coated with target-specific antibodies, secondary antibodies, proteins, or any molecular entity of interest. A signal is produced when the AS Acceptor and Donor beads are brought into proximity (<200 nm) by a molecular interaction occurring between the binding partners captured on the beads. Laser excitation of the Donor beads at 680 nm causes ambient oxygen to be converted to the singlet state by photosensitizers (phthalocyanine). These react with chemiluminescent agents (thioxene, anthracene, rubrene) within the Acceptor bead only when the latter is in close proximity (Fig. **2**). Upon energy transfer between those compounds, activated rubrene emits light at 520-620 nm which is in turn detected by the photodetector in a microplate reader. An excitation wavelength higher than the emission wavelength ensures a low assay fluorescent background by avoiding any auto-fluorescence from biological media or compounds. However, AS may be sensitive to other types of interferences. Antioxidants or other quenchers of reactive oxygen species like metal ions can strongly affect the emitted signal. Moreover, since the AS detection is only based on a fluorescence-intensity measurement, colored compounds absorbing in the 500–600 nm wavelength range artificially decrease the AS signal and therefore may be detected as false positives in HTS. To circumvent these limitations, Acceptors beads were modified to contain Europium. Europium (Eu) has a long fluorescent lifetime, longer than several hundreds of microseconds, whereas traditional organic reagents have several nanoseconds; the emission peaks of Eu complexes are 615 nm and the fluorescent peak profiles are sharp: the half-widths are 10 nm – 20 nm. It is known that the fluorescence is based on the energy transfer from the ligand to the central metal ion. The characteristics of these new beads allowed for the development of a novel assay format commercialized as AlphaLISA™. Using such fluorescent properties this assay leads to the reduction of background interferences of short lifetime from the sample matrix, and enables highly sensitive detection and thus leads to greatly increased specificity and sensitivity of the assay.

AlphaScreen™ is a bead-based non-radioactive and homogeneous detection technology. Donor and Acceptor microbeads can be coated with target-specific antibody, proteins, or secondary reagents (streptavidin, glutathione, Nickel). A signal is produced when the AlphaScreen™ Acceptor and Donor beads are brought into proximity by a molecular interaction occurring between the binding partners captured on the beads. Laser excitation at 680 nm causes ambient oxygen to be converted to the singlet state by photosensitizers on the Donor bead. These react with chemiluminescent agents on the Acceptor bead only when the latter is in close proximity, emitting light at 520-620 nm. AlphaScreen™ allows one to quantify various analytes by performing competition assays and interpolating signals with a standard curve.

## 3). HOW DOES AS CONTRIBUTE TO DECIPHERING CELL SIGNALING PATHWAYS?

In the following sections, we will extensively review the literature and explore the use of AS as a tool to explore cell signaling pathways. The scheme of our analysis is shown on Fig. (**3**). Briefly, we will describe the AS-based assays monitoring the interaction between receptors and their respective ligands, then those aiming at evaluating the activation of specific signaling pathways by measuring the intracellular concentrations of second messengers, enzymatic activities, or specific sub-cellular localization and finally the assays which aim at measuring the occurrence of specific post-translational modifications whose role could be involved in various aspects of cell signaling.

### a). Receptor-Ligand Interactions

#### i). G Protein-Coupled Receptors

Thus far, only two types of assays have been developed to evaluate receptor-ligand interactions. The first type of assay aims at characterizing the human GPR3, GPR6 and GPR12 receptors which belong to the GPCR superfamily. These proteins exhibit high similarity to each other. Devoid of any identified natural ligand, these receptors are currently classified as orphan GPCRs [3]. In an attempt to characterize their activation process, various functional AS-based assays (cAMP AS assays, mobilization of intracellular Ca^2+^, agonist-induced internalization) were applied to demonstrate that Sphingosine 1-phosphate (S1P) and dihydrosphingosine 1-phosphate (DHS1P) were *bona fide* activators of the GPR3, GPR6 and GPR12 family [4]. Lysophosphatidic acid (LPA, 1-acyl-2-*sn*-glycerol-3-phosphate) signaling through G proteins mediates a variety of biological functions including cell proliferation, cell survival, cytoskeleton remodeling, cell migration, and alterations in differentiation [5, 6].

Five LPA-specific GPCRs have thus far been identified, termed LPA_1–5_ [7-9]. LPA_4,_ also known as GPR23, is the only receptor that has yet to receive independent confirmation as a *bona fide* LPA receptor since its initial report [9]. This putative LPA receptor particularly stands out owing to its relatively low predicted amino acid sequence homology compared with the well studied LPA_1–3_, a *K_d_* ~45 nM for LPA, and an ability to mobilize calcium and increase cAMP production. Recently, Lee and colleagues confirmed the finding that GPR23 is indeed a biologically relevant receptor for LPA and report several novel aspects of LPA_4_ signaling that extend its functional roles [8].

#### ii). TNFalpha Receptor

Wilson and colleagues developed an AS-based high-throughput assay to monitor ligand binding to a member of the tumor necrosis factor (TNF) receptor superfamily [10]. The binding affinity of the extracellular domain of the OX40 receptor (fused to the constant domains of human IgG) to the OX40 ligand was determined. In addition, using the same approach small peptides capable of inhibiting the OX40 receptor: ligand interaction were also identified.

### b). Studying GPCR-Dependent Signal Transduction Pathways

The signaling pathways which have been the most studied using AS are those downstream of GPCR activation. The GPCR superfamily constitutes a large group of membrane receptors known to modulate a wide range of biological responses, including cell growth, differentiation, migration, and inflammatory processes [11]. The initial stage of GPCR activation occurs *via* agonist-induced conformational changes in the receptor to form an active agonist–receptor complex that is able to interact with heterotrimeric G-proteins and facilitate its activation *via* exchange of GDP for GTP at the α-subunit. All known effectors are regulated by the dissociated α or βγ subunits and not by the heterotrimer. Effectors include mainly enzymes such as adenylyl cyclases, phospholipases, phosphodiesterases, or kinases and ion channels.

#### i). Monitoring cAMP Levels

Production of cAMP is controlled through the adenylyl cyclase family of enzymes, which convert adenosine triphosphate (ATP) to cAMP and inorganic pyrophosphate. These enzymes are activated or inhibited *via* direct interaction with G protein α-subunits and, for some isoforms, with Ca^2+^ and calmodulin. Following Gs-coupled GPCR activation, active Gαs molecules exert a positive effect on adenylyl cyclase catalysis; cAMP is produced and is then able to bind to protein kinases within the cell leading to the regulation of target enzymes and transcription factors [12, 13].

Changes in the levels of intracellular cAMP can be measured directly through competition of antibody-captured labelled cAMP, with demonstrated assay-specific limitations [14, 15]. An alternative high-throughput homogeneous platform assay has been developed for screening GPCRs which is based on the measurement of intracellular cAMP concentrations [15-19]. Gabriel and colleagues reported a direct comparison of AlphaScreen™, HTRF, HitHunter™ and FP cAMP assay platforms and suggest the use of AS or HTRF for cells expressing low levels of GPCRs owing to higher sensitivities [14]. As an example, parathyroid hormone (PTH), a major regulator of bone remodeling and calcium ion homeostasis, exerts its effects by binding and activating the GPCR superfamily member PTH/PTH-related peptide receptor (PPR). Carter and Schipani showed that a modified synthetic peptide (SW106) equivalent to PTH fragment (1–34) had PPR antagonistic effects determined using AS [20]. The ability of SW106 to block PTH-initiated cAMP accumulation was shown to be specific to its actions on the PPR and not related to its ability to interfere with other signaling mechanisms.

### c). Monitoring Lipid Signaling

Many cellular responses elicited by GPCRs are mediated by phospholipid signaling cascades, initiated by Gα and Gβγ subunits of heterotrimeric G proteins. The hydrolysis of membrane phospholipids leads to the formation of various bioactive lipid mediators acting either as extracellular signaling molecules or as intracellular second messengers.

A crucial second messenger-generating system is the stimulation of phosphoinositide-specific phospholipase C (PLC) isoforms. Upon activation, PLC enzymes hydrolyze phosphatidylinositol-4,5-bisphosphate (PIP_2_) at the inner face of the plasma membrane and thereby generate the messengers, diacylglycerol (DAG) and inositol-1,4,5-trisphosphate (IP_3_).

#### i). PI-3 Kinase

Gray and colleagues [21], using suitably tagged pleckstrin homology (PH) domains as probe and the AS technology or time-resolved FRET, quantified phosphoinositides in cell extracts. DAG and IP3 signaling molecules led to the activation of several protein kinase C (PKC) isoforms and the release of calcium from intracellular stores, respectively [21].

Phosphoinositide 3-kinases (PI3Ks) represent a family of dual specificity enzymes that can act as lipid and protein kinases to regulate numerous biological processes including cell growth, differentiation, survival, proliferation, migration and metabolism [22, 23]. The lipid kinase activity of PI3K catalyzes the addition of a phosphate group at the D-3 position of phosphatidylinositol lipids, generating different 3' phosphorylated products that act as second messengers [24].

The regulatory subunit of PI3Ks – p85 – harbors a p110-binding region flanked by two SH2 (Src Homology 2) domains, which are pivotal in mediating the activation of class IA PI3Ks by receptor tyrosine kinases (RTK). Indeed, SH2 domains of the p85 protein specifically bind to phosphotyrosine residues in the YXXM motif on the cytosolic tails of receptor tyrosine kinases or other membrane-associated proteins, eventually docking the holoenzyme adjacent to the plasma membrane phospholipid bilayer, where its lipid substrates reside [23]. Moreover, the PI3K catalytic subunit p110β harbors the ability to be synergistically triggered by both G proteins and phosphotyrosyl peptides and might thus function by integrating signals from both GPCR- and RTK-signaling cascades.

Different types of phosphatidylinositol 3-phosphates can be generated by PI3Ks. *In vivo*, class I kinases predominantly produce PtdIns(3,4,5)P3 from PtdIns(4,5)P2, while class II and III enzymes mainly convert PtdIns to PtdIns(3)P. PtdIns(3,4,5)P3 exerts its second messenger function by recruiting and activating a wide array of proteins harboring a PH domain (PDK1, PLCγ, GEFs and GAPs), which in turn initiate numerous intracellular responses. PH domains represent the best characterized elements binding PIP2 and PIP3. They exist as a large consensus sequence domain family that includes diverse members differing in their ability to bind to distinct phosphoinositides; whereas the PH domain found in PKB/AKT, BTK, and PDK1 recognize PtdIns (3,4,5)P3 with high affinity and specificity, others such as those found in PLCδ, TAPP1 and TAPP2 interact exclusively with PtdIns-(4,5)P2 [25]. Among the PH domain-containing proteins activated by PtdIns(3,4,5)P3, of particular interest are phosphoinositide-dependent kinase 1 (PDK1) and the serine/threonine kinase PKB/AKT [26], both integral components of key cellular signaling pathways.

A novel approach to quantitation of phosphoinositides in cell extracts and in *in vitro* enzyme-catalyzed reactions has been described and consists in using labeled pleckstrin homology (PH) domain probes [21]. PtdIns(3,4,5)P3 present in lipid extracts derived from Swiss-3T3 and HL60 cells stimulated with platelet-derived growth factor or with the bacterially-derived peptide fMLP (formyl-methionyl-leucyl-phenylalanine), respectively, was shown to be detectable at picomole sensitivity; stable complexes were generated between the biotinylated target lipid and the appropriate PH domain, and phosphoinositides present in samples were detected by their ability to compete for binding to the PH domain. Complexes were detected using AS technology or time-resolved FRET. Interestingly, the elevated flexibility of this assay platform was evaluated by swapping GRP1 and TAPP1 PH domains which respectively bind exclusively to PIP3 and PIP2.

PDK1-mediated activation of AKT, the key effector of class I PI3K signaling, results in modulation of distinct signaling cascades regulating cell proliferation (GSK3, FOXO), survival (BAD, MDM2) and protein synthesis/cell growth (TSC, RHEB, mTOR). One example of the above-mentioned PH domain competition approach is the development of a kit that would monitor the presence of the phosphoinositide PIP3, a second messenger in the activation pathway of AKT kinase; upon activation of PI3-kinase, PIP3 is produced which activates PDK1. PDK1, in turn, activates AKT by phosphorylation at the Thr308 residue. Consequently, the level of PIP3 would directly reflect PI3K activity and potentially link it to the activation level of AKT.

### d). GTPase Signaling

The superfamily of small GTPases constitutes an essential component of the signaling pathways that link extracellular signals *via *transmembrane receptors to cytoplasmic or nuclear responses. The Ras-superfamily of small GTPases and their G-protein cousins act by a conserved mechanism: the molecules' signaling activities differ when bound to GTP versus GDP, and their GTPase activity (GTP hydrolysis to GDP) determines their GTP-bound versus GDP-bound state. In the development of a new assay allowing the systematic comparison of the biochemical properties of monomeric GTPases of the Ras superfamily. Caruso and colleagues used two high-throughput methods, AS and FlashPlate^®^, to measure nucleotide binding capacity, nucleotide exchange, and GTP hydrolysis activities of small monomeric GTPases; integration of the results from this study led to the proposal of a novel classification of Rho GTPases based on their enzymatic activities [27].

### e). Monitoring Protein Kinase Activity

The emergence of technologies allowing high-throughput, system-wide experiments has provided a detailed and objective view of downstream transcriptional changes following various cellular stimuli. However, many critical events involved in cellular responses are mediated by changes in post-translational protein modifications rather than transcriptional changes. It is well established that protein modifications can influence and control enzymatic activity, protein conformation, protein:protein interactions, and cellular localization. Even for protein phosphorylation, which affects an estimated one-third of all proteins and is the most widely studied post-translational modification [28], only a small subset of total *in vivo* phosphorylation sites has been discovered so far [29].

Protein kinases represent one of the most important classes of therapeutic targets in high-throughput drug screening. Tyrosine kinases and serine/threonine kinases are known to play key roles in signal transduction as well as in cell growth and differentiation. Recently, homogeneous protein kinase assays including fluorescence resonance transfer assays (FRET) [30], fluorescence polarization assays and AS assays [31-35], ATP consumption assays and superquenching assays [36] have been developed for the HTS of kinase activity. Indeed, the use of such assays in HTS, and probably a combination of them, may allow the detection of a broader spectrum of inhibitors presenting higher specificities.

#### i). JNK Signaling

The c-Jun N-terminal protein kinases (JNK) are encoded by three genes: while *Jnk1* and *Jnk2* genes are expressed ubiquitously, *Jnk3* has a more limited pattern of expression and is largely restricted to brain, heart, and testis. Remarkably, these genes are alternatively spliced to create ten JNK isoforms which, together with ERK and p38^MAPK^, are implicated in various MAP kinase-dependent pathways. The JNK pathway has been implicated in both apoptosis and survival signaling, and is activated by exposure of cells to stress stimuli (e.g. UV). To model ocular diseases of the retina (such as ESCS (Enhanced S-Cone) syndrome) and photoreceptor degeneration (RPE65^-/-^), RPE (retinal pigment epithelium) cells were exposed to UV irradiation, and JNK protein expression was monitored by an AS-based assay [37]. The activation (phosphorylation) of JNK kinases (1, 2, 3) upon varying treatment doses and duration of UV irradiation was observed. The activator protein-1 (AP-1) was itself activated through phosphorylation of c-Jun and c-Fos, induced by JNK and p38, respectively. Moreover using various isoforms of JNK inhibitory peptides (long, short and D- forms; JNKi) it seems that UV-induced JNK activation reduced pro-survival effects of ERK2 which was reversed by pre-incubation with the JNKi peptide (retro-inverso peptide). Using the same luminescence-based approach Guenat and colleagues identified a peptide inhibitor and characterized its mechanism of action by measuring its binding affinity for endogenous JNK [31].

#### ii). JAK Signaling

The family of signal transducers and activators of transcription (STATs) consists of seven transcription factors that respond to a variety of cytokines, hormones, and growth factors. STATs are activated by tyrosine phosphorylation, which results in their dimerization and translocation into the nucleus where they exert their effect on transcription of regulated target genes. The phosphorylation of STATs is mediated mainly by Janus kinases (JAKs), one of ten recognized families of non-receptor tyrosine kinases. Mammals have four members of this family, JAK1, JAK2, JAK3 and Tyrosine kinase 2 (TYK2).

The JAK/STAT pathway plays a critical role in hematopoietic and immune cell function. In particular, STAT5 is phosphorylated by several JAKs, including Jak3, JAK2, and TYK2, in response to interleukin-2, erythropoietin (EPO), and interleukin-22, respectively. Using the SureFire pSTAT5 assay that utilizes AS, Binderand and colleagues evaluated EPO-induced STAT5 phosphorylation in HEL cells and successfully completed a small-scale screening campaign to identify inhibitors of this biological event [38].

In addition, the suppressor of cytokine signaling (SOCS) which is involved in the negative regulation of the cytokine-induced JAK/STAT pathway was studied using AS [39]. In order to decipher whether SOCS2 is a major negative regulator of GH action, Greenhalgh *et al*., (2005), using SPR technology, characterized the interaction between SOCS2 and the GH receptor and found that SOCS2 binds to 2 phosphorylated tyrosines on the GH receptor. An AS-based interaction assay indicated that phosphorylated Tyr595 and Tyr487 peptides derived from the GH receptor were able to bind to the SOCS2 SH2 domain, which was confirmed *in vivo* by their ability to stimulate the STAT5 pathway [40].

#### iii). ERK Signaling

Detection of the activation of ERK1/2 in Gq-coupled GPCR systems was demonstrated using AS-based methodologies and led to comparable pharmacological data for receptor agonist and antagonist data obtained using GPCR activation measurement techniques [41]; whether amino acids mediate the activation of ERK1/2 is nevertheless still poorly documented. Lee and colleagues examined the effect of L-amino acids on Ca^2+^-stimulated ERK1/2 phosphorylation by Western blotting and the SureFire AS-based phosphorylated ERK1/2 assay [8]. By both these methodologies, these authors demonstrate that certain amino acids allosterically activate extracellular Ca^2+^-stimulated ERK1/2 phosphorylation in Ca^2+^ sensing Receptor expressing HEK-293 cells [8].

### f). Other Types of Signaling-Related Information

#### i). Monitoring Nuclear Localization

**Fig. (3). Schematic representation of a canonical signaling pathway “from the plasma membrane to the nucleus”.**
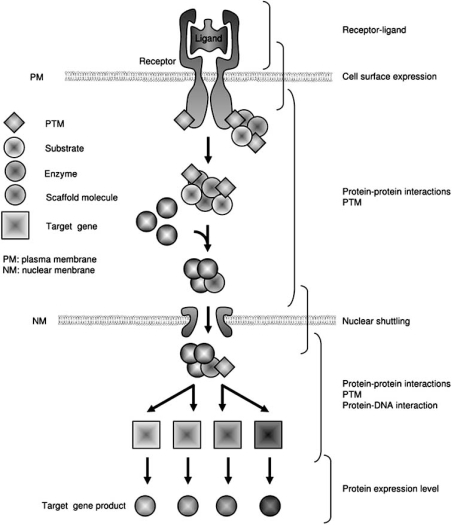
The receptor-ligand interaction occurring in the extracellular space leads to the activation of the membrane receptor which transduces a signal into the cytoplasm. At this stage, signaling can involve post-translational modifications of proteins (PTM), formation of complexes (scaffolds), modification of lipids and enzyme cascade activation (GTPases, kinases). Activated complexes can then translocate in the nucleus where transcriptional activation processes can take place, followed by mRNA maturation, expression and translation into active proteins.

An important feature in the completion of signaling pathways from the plasma membrane to the nucleus is the nuclear translocation steps, which brings in contact genomic DNA and potential regulators of its expression, structure etc (Fig. **3**). This occurs when cargo molecules containing specific nuclear localization sequences (NLS) bind to karyopherins, such as importin-β and importin-α, to form complexes (importin-β/importin-α/cargo) that are transported into the nucleus through interactions with nucleoporins in the nuclear pore complex NPC. Hearps and Jans reported that the integrase, known to be also involved in the nuclear import of HIV DNA, exhibits a high affinity interaction with importin-α and with the heterodimer α/β  [42]. Similarly, using an AS-based assay and recombinant Hisx6 and biotin-tagged proteins, Walgstaff and Jans determined the high affinity of the importin-α/β heterodimer to the NLS-containing protein of SV40 T antigen and HIV integrase. A similar assay was performed to define flanking residues implicated in the modulation of the IMP-NLS interaction [43]. In a recent paper, Bartholomeeusen and colleagues verified the specific interaction between JPO2 and LEDGF/p75 by pull-down, AS [44], which was also confirmed by co-immunoprecipitation [45]. Moreover, in AS competition assays, the HIV-1 integrase showed a mutually exclusive binding to either JPO2 or LEDGF/p75. However, differing mechanisms of binding were suggested by continuing interaction of JPO2 with some LEDGF/p75 mutants that are totally defective for interaction with HIV-1 integrase [46,47]. In another study, Al Mawsawi and colleagues observed an inhibitory effect of the LEDGF peptide on the LEDGF/P75-interaction (his6-tagged integrase and flag-LEDGF/P75) [48].

#### ii). Nuclear Receptors

Nuclear receptors are known to function as multimeric structures and generally show a large spectrum of ligands. In this section, we will illustrate how the AS technology was used to monitor the functionality of four different nuclear receptors. The first example is provided by the constitutive androstane receptor (CAR; NR1I3) which is an orphan nuclear receptor that is predominantly expressed in the liver [49, 50]. CAR and Pregnane X receptor (PXR) share a highly conserved DNA binding domain (DBD). Both receptors bind to DNA as a heterodimeric complex with RXR, which binds to the vitamin A metabolite 9-*cis*- retinoic acid (9cRA). In addition to CAR and PXR, RXR also serves as an obligate heterodimeric partner for about fifteen other nuclear receptors including receptors for retinoic acid (RAR), thyroid hormone (TR), vitamin D (VDR), and fatty acids (PPARs). Binding of the TIF2 (transcription intermediary factors member of the p160 family of nuclear receptor coactivators) motif and ligands to CAR or CAR/RXR heterodimers were determined using AS. These experiments were conducted with Ligand Binding Domain receptor and biotinylated TIF2 peptide, the IC_50_ was determined for the CAR activator TCPOBOP (1,4 bis[2-(3,5-dichloropyridyloxy)]benzene) and androstanol [51].

The functional interaction between the orphan nuclear receptors, small heterodimer partner (SHP) and liver receptor homolog 1 (LRH-1), where SHP binds to LRH-1 and represses its constitutive transcriptional activity, is crucial for regulating genes involved in cholesterol homeostasis. Li and colleagues reported structural and biochemical analyses of the LRH-1-SHP interaction, and the binding of the cofactor motifs to the nuclear receptors was determined using AS [32]. They demonstrated that only the second SHP LXXLL motif is required for repressing LRH-1, and that this motif displays a strong preference for binding to LRH-1 over the closely related receptor steroidogeneic factor 1 (SF-1).

Finally, the herbicide Atrazine (ATR) binds directly to SF-1 and induces the cAMP phosphodiesterase (PDE) 4D, which is expressed in human fetal tissues and is proposed to mediate inflammatory responses in the myometrium. ATR response seems to be mediated *via* convergence of NR5A (nuclear receptor family) activity and cAMP signaling, to potentially disrupt normal endocrine development and function. Using cellular and biochemical assays several lines of evidence suggest that ATR might not function by directly binding to NR5A receptors and failed neither to alter coactivator peptide recruitment using an AS assay nor to displace the bacterial phosphatidyl glycerol or an exchanged PIP3 ligand present in the SF-1 ligand binding pocket [52]. ATR also activates PI3K signaling as evidenced by the increase in phosphorylation of AKT or protein kinase B.

#### iii). Protein Metabolism

The best example of protein modification, characterization and enzyme substrate interaction was reported previously [53]. In this study, the authors attempted to identify specific substrates of the still uncharacterized E3 Ubiquitin-protein ligase RSP5. Using AS to detect ubiquitination *in vitro*, the authors screened hundreds of purified yeast proteins for ubiquitination, and identified previously reported and novel substrates of the yeast E3 ligase RSP5. The relevance of these substrates was confirmed *in vivo* by showing that a number of them interact genetically with Rsp5, and some were ubiquitinated by RSP5 *in vivo*.

## 4). PERTURBING SIGNALING PATHWAYS

This section aims at reviewing all the AS-based approaches that successfully identified compounds, small molecules, or peptides whose biological activity was to perturb specific signaling pathways. On the one hand, we will review the literature available and on the other hand describe the results available in public repositories.

### a). Literature Review

Several examples of the use of AS in the identification of signaling pathway modulators are available in the literature. The first type of assays which was used to identify such molecules was based on monitoring the activation levels of second messengers downstream of membrane receptors in response to agonists or antagonists. This is illustrated for instance by the Human formyl peptide-receptor-like-1 (FPRL-1) which is a promiscuous G protein-coupled receptor (GPCR), and belongs to a chemo-attractant receptor family protein. The FPRL-1 mediated release of cAMP was measured using AS. To stimulate cAMP production, forskolin was added at 10 *μ*M (final concentration) to HEK293 cells. The concentrations of ligands tested were 10 pM to 200 nM for chemokines, 1 pM to 20 nM for W-peptide and 0.5 nM to 10 *μ*M for other compounds and a potent agonist of FPRL-1 was identified as being a truncated form of CKbeta8-1 [54]. Similarly, Uhlenbrock and colleagues [4] discovered GPCR activators (sphingophospholipids) and Bley and colleagues reported the characterization of structurally distinct, potent and selective IP (prostacyclin) receptor antagonists using the same type of assay [55].

The second type of assays developed to identify agents perturbing signaling pathways is based on the evaluation of altered protein-protein interactions. This was illustrated for the study of Hepatocyte Growth Factor receptors. Indeed as receptor dimerization is a general paradigm for activation of RTKs, Tolbert *et al*. (2007) exploited AS technology to evaluate binding affinity of human NK1, a natural variant of hepatocyte growth factor (HGF), to Met receptor tyrosine kinase [56]. The results suggested that NK1 was capable of binding and inducing Met dimerization in a heparin-dependent manner which was confirmed *in vivo*. Similarly and at a more general level, the use of the AS technology was proposed to study the concept of altered protein-protein interactions in the identification of perturbing agents. Indeed as protein-protein interactions allow the recruitment of cytoplasmic polypeptides to activated receptors, direct their assembly into larger complexes, target them to defined subcellular locations, and determine the specificity with which enzymes interact with their substrates, they consequently represent targets of choice to be perturbed [57].

### b). Public Repositories

In addition to the published data mentioned in the previous section, a repository of the AS-based results obtained in the screening of compound libraries is available at NCBI (http://www.ncbi.nlm.nih.gov/) in the PubChem Bioassay database [58] which contains bioactivity screens of chemical substances described in PubChem Substance. It provides searchable descriptions of each bioassay, including descriptions of the conditions and readouts specific to that screening procedure. At present, eight AS-based screens are reported which aim at identifying inhibitors of Her-Kinase Expression (AID: 461, AID: 742, AID: 645, AID: 814), inhibitors of ERK1 signaling (AID: 995), inhibitors of JNK3 signaling (AID: 530), and finally disruptors of Hsp90 Co-chaperone interaction (AID: 632, AID: 595).

## 5). CONCLUSIONS AND PERSPECTIVES

In the above sections, we have described how AS was use to monitor, dissect and perturb the molecular mechanisms playing a role in the activation of specific signaling pathways.

It is clear that each component of a given signaling pathway could be analyzed individually at the genome scale (for instance analyzing kinomes, interactomes, etc.) however the integrated representation of a number of signaling pathways activated in response to specific stimuli would definitely provide a better understanding of the molecular mechanisms set in place in the cell to respond.

Moreover, it is interesting that most of the signaling steps described in Fig. (**3**) were evaluated using a unique technological approach as AS. This most likely reflects the high versatility of the technology. In addition, although most of the assays described in this review have demonstrated experimental robustness, sensitivity, and efficiency, they were carried out separately and despite of the objectives of providing a complete and integrated view of the signaling pathways activated in response to given stimuli.

Currently, with the increasing needs from both industry and academia to evaluate the global impact of various challenges on cells signaling pathways, it is easy to anticipate that integrated platforms allowing for global (and as exhaustive as possible) analyses of signal transduction pathways in parallel and in formats compatible with high-throughput screening will emerge.

The establishment of such tools will be of course facilitated by the use of a single technology adaptable to the evaluation of an almost infinite number of parameters. In this context, the problematic would necessarily locate beyond the development of the assays, but rather at the level of the interpretation, integration and visualization of the quantitative data.
